# Cancer Specific Long Noncoding RNAs Show Differential Expression Patterns and Competing Endogenous RNA Potential in Hepatocellular Carcinoma

**DOI:** 10.1371/journal.pone.0141042

**Published:** 2015-10-22

**Authors:** Jian Zhang, Dahua Fan, Zhixiang Jian, George G. Chen, Paul B. S. Lai

**Affiliations:** 1 Department of Surgery, The Chinese University of Hong Kong, Prince of Wales Hospital, Shatin, New Territories, Hong Kong SAR, China; 2 Division of Gastrointestinal Surgery & Gastric Cancer Center, The First Affiliated Hospital of Sun Yat-sen University, Guangzhou, Guangdong, China; 3 The First Affiliated Hospital of Shenzhen University, Shenzhen, Guangdong, China; 4 Department of General Surgery, Guangdong General Hospital, Guangdong Academy of Medical Sciences, Guangzhou, Guangdong, China; The University of Hong Kong, CHINA

## Abstract

Long noncoding RNAs (lncRNAs) regulate gene expression by acting with microRNAs (miRNAs). However, the roles of cancer specific lncRNA and its related competitive endogenous RNAs (ceRNA) network in hepatocellular cell carcinoma (HCC) are not fully understood. The lncRNA profiles in 372 HCC patients, including 372 tumor and 48 adjacent non-tumor liver tissues, from The Cancer Genome Atlas (TCGA) and NCBI GEO omnibus (GSE65485) were analyzed. Cancer specific lncRNAs (or HCC related lncRNAs) were identified and correlated with clinical features. Based on bioinformatics generated from miRcode, starBase, and miRTarBase, we constructed an lncRNA-miRNA-mRNA network (ceRNA network) in HCC. We found 177 cancer specific lncRNAs in HCC (fold change ≥ 1.5, P < 0.01), 41 of them were also discriminatively expressed with gender, race, tumor grade, AJCC tumor stage, and AJCC TNM staging system. Six lncRNAs (CECR7, LINC00346, MAPKAPK5-AS1, LOC338651, FLJ90757, and LOC283663) were found to be significantly associated with overall survival (OS, log-rank P < 0.05). Collectively, our results showed the lncRNA expression patterns and a complex ceRNA network in HCC, and identified a complex cancer specific ceRNA network, which includes 14 lncRNAs and 17 miRNAs in HCC.

## Introduction

Noncoding RNAs are RNA molecules that are not coding for proteins. They can be divided into several subtypes including long non-coding RNA (lncRNA), microRNA (miRNA), ribosomal RNA (rRNA), small nucleolar RNA (snoRNA), and transfer RNA according to HUGO Gene Nomenclature Committee (HGNC) (http://www.genenames.org).

After the identification of lncRNA in malignancy diseases, an increasing number of studies on the biological roles of lncRNAs have been conducted in various cancers, including HCC [1], esophageal squamous cell carcinoma [2], colorectal cancer [3], renal cell carcinoma [4] and prostate cancer [5]. The abnormal expression of lncRNAs through interactions with miRNAs or mRNAs is involved in the regulation of tumor progression and tumor biological behaviors in HCC [6–8]. The cancer specific lncRNAs may also impact the invasion and metastasis of HCC [9].

In 2011, Salmena *et al*. presented a competing endogenous RNA (ceRNA) hypothesis, which unified the transcriptome and formed a regulatory RNA network [10]. The main idea is that all types of RNA transcripts communicate with each other by competing for binding to shared miRNA-binding sites (“miRNA response elements” or “MREs”). This kind of RNA competition crosstalk exists between protein-coding messenger RNAs and non-coding RNAs such as lncRNA, pseudogenes and circular RNAs [11]. Furthermore, artificial miRNA sponges can also participate in this network to regulate gene expression [12].

Zhu et al. reported that the lncRNA expression profile of HCC by microarray analysis from three HCC patients [13]. However, there is lack of studies with large scale sample size and high through detection methods on the expression patterns of cancer specific lncRNA in HCC, and it is unknown whether lncRNAs are correlated with overall survival, gender, or other clinical features or whether the aberrant expression of lncRNAs in HCC has any ceRNA potential. Recently, RNA sequencing data from The Cancer Genome Atlas (TCGA) project or GEO provide the public with lncRNA, miRNA, and mRNA data for HCC. To address the above mentioned questions, we explored lncRNAs in HCC using data sets from TCGA and GEO. These two data sets included RNA sequence results for a total of 372 HCC tumor tissues and 48 adjacent non-tumor liver tissue samples. To the best of our knowledge, this study is the first to make use of large scale sequencing database to investigate the cancer specific lncRNA expression patterns and ceRNA network in HCC. This new approach of predicting cancer specific lncRNA and ceRNA network can help us to understand the function of lncRNAs in HCC.

## Methods

### Patients and samples

A total of 360 patients with HCC were retrieved from the TCGA data portal. The exclusion criteria were set as follows: 1) histologic diagnosis is not HCC; 2) samples without completed data for analysis; and 3) Overall survival more than 2000 days. Overall, a total of 322 HCC patients were included in our study. Among these 322 HCC patients, the adjacent non-tumor liver tissues were retrieved from 43 subjects. This study meets the publication guidelines provided by TCGA (http://cancergenome.nih.gov/publications/publicationguidelines). Another GEO data set (GSE65485) was downloaded from GEO (http://www.ncbi.nlm.nih.gov/geo/) which included 50 HCC tissues and 5 adjacent non-tumor liver tissues. As the data were obtained from TCGA and GEO, further approval by an ethics committee was not required.

### RNA sequence data procession and computational analysis

The RNA expression data (level 3) of the corresponding patients (tumor and/or adjacent non-tumor tissues) were downloaded from TCGA data portal (up to Feb 24, 2015). The lncRNA and mRNA expression profiles were generated from RNA sequencing raw reads by RNASeqV2 post-processing pipelines and demonstrated as RSEM (RNA-Seq by Expectation-Maximization) normalized count data. The miRNA expression profile was performed using the Illumina HiSeq 2000 miRNA sequencing platforms (Illumina Inc, USA) and demonstrated as reads per million miRNA (RPM) mapped data. Because the mRNA, lncRNA, and miRNA expression profile data were already normalized by TCGA, no further normalizations were applied to these data. The GEO data set was also generated from the Illumina HiSeq 2000 platform and normalized as FPKM (fragments per kilo bases of exons for per million mapped reads) data. The lncRNA analyses were performed using BRB-ArrayTools (version 4.4) developed by Dr. Richard Simon and the BRB-ArrayTools Development Team [14].

### Construction of the ceRNA network and KEEG Pathway Analysis

The construction of ceRNA network included three steps: (i) cancer specific lncRNA filtration: cancer specific lncRNAs with absolute fold change ≥ 3.0 (either up-regulation or down-regulation) and P < 0.05 were retained. To improve the data reliability, cancer specific lncRNAs have not been annotated by GENCODE (http://www.gencodegenes.org/) were discarded; (ii) lncRNA-miRNA interactions were predicted by miRcode (http://www.mircode.org/) and starBase v2.0 (http://starbase.sysu.edu.cn/); (iii) mRNAs targeted by miRNAs with experimental support were retrieved from miRTarBase (http://mirtarbase.mbc.nctu.edu.tw/). To further enhance the robust of this ceRNA network, the maximal information coefficient (MIC) algorithm and maximal information-based nonparametric exploration (MINE) statistics were used in TCGA data set to filter the pair-wised relationships [15]. A network graph was constructed and visualized using Cytoscape v3.0 [16]. The coding genes involved in ceRNA network were input into the Database for Annotation, Visualization and Integrated Discovery (DAVID) [17] for KEEG pathway enrichment analysis.

### Statistical analysis

Data were presented as mean ± SD. Differences among groups were evaluated by two-sample t test and the significance level was set as 0.001 as default to control the false discovery rate (FDR). The univariate Cox proportional hazards regression was conducted to find out the lncRNAs correlated with overall survival [18]. P value less than 0.05 was considered as statistical unless specifically indicated. The statistical analyses were performed by BRB-ArrayTools or R language [19].

## Results

### Cancer specific lncRNAs in HCC

We identified 604 lncRNAs from TCGA data set and 357 lncRNAs from GSE65485. For any single lncRNA, it appeared in TCGA data set or both TCGA and GEO data sets. We found that 177 lncRNAs and 37 lncRNAs were differentially expressed between HCC tissues and adjacent non-tumor tissues in TCGA data set and GEO data set (absolute fold change ≥ 1.5, P < 0.01, S1 Table), respectively. To further enhance the data reliability, we selected 28 lncRNAs included in GENCODE and these lncRNAs had an absolute fold change ≥ 3.0 from either TCGA or GEO data set to build ceRNA network [20]. Finally, 28 lncRNAs (18 up-regulated; 10 down-regulated) were selected for ceRNA network (Table 1).

**Table 1:** Twenty eight cancer specific lncRNAs in ceRNA network construction. This table showed 28 cancer specific lncRNAs for ceRNA network construction with absolute fold change ≥ 3.0, P < 0.01 and included in GENCODE.

LncRNA	Gene ID	Chromosome	Expression change (T vs. N)	Data set
ASMTL-AS1	ENSG00000236017	chrX	Up-regulation	TCGA
CDKN2B-AS1	ENSG00000240498	chr9	Up-regulation	TCGA + GEO
CECR7	ENSG00000237438	chr22	Up-regulation	TCGA
FLVCR1-AS1	ENSG00000198468	chr1	Up-regulation	TCGA
FOXD2-AS1	ENSG00000237424	chr1	Up-regulation	TCGA
GAS5	ENSG00000234741	chr1	Up-regulation	TCGA
IGF2BP2-AS1	ENSG00000163915	chr3	Up-regulation	TCGA
LINC00152	ENSG00000222041	chr2	Up-regulation	TCGA
LINC00176	ENSG00000196421	chr20	Up-regulation	TCGA
LINC00488	ENSG00000214381	chr3	Up-regulation	TCGA
LINC00685	ENSG00000226179	chrX	Up-regulation	TCGA
PVT1	ENSG00000249859	chr8	Up-regulation	TCGA + GEO
RUSC1-AS1	ENSG00000225855	chr1	Up-regulation	TCGA
SNHG1	ENSG00000255717	chr11	Up-regulation	TCGA
SNHG3	ENSG00000242125	chr1	Up-regulation	TCGA + GEO
SNHG4	ENSG00000281398	chr5	Up-regulation	TCGA + GEO
TSPEAR-AS2	ENSG00000182912	chr21	Up-regulation	TCGA
ZNF252P-AS1	ENSG00000255559	chr8	Up-regulation	TCGA
DIO3OS	ENSG00000258498	chr14	Down-regulation	GEO
FAM99A	ENSG00000205866	chr11	Down-regulation	TCGA + GEO
FAM99B	ENSG00000205865	chr11	Down-regulation	TCGA
H19	ENSG00000130600	chr11	Down-regulation	TCGA
HAND2-AS1	ENSG00000237125	chr4	Down-regulation	TCGA
HAR1A	ENSG00000225978	chr20	Down-regulation	TCGA
LINC00238	ENSG00000196553	chr14	Down-regulation	TCGA
LINC01554	ENSG00000236882	chr5	Down-regulation	TCGA
PWRN1	ENSG00000259905	chr15	Down-regulation	GEO
UCA1	ENSG00000214049	chr19	Down-regulation	TCGA

### The correlations between cancer specific lncRNAs and clinical features

The 177 lncRNAs from the above section were further analyzed according to clinical features including gender, race, tumor grade, AJCC TNM staging system, AJCC pathological stage, vascular invasion, new tumor event, tumor status, and age at diagnosis in TCGA and/or GEO data sets. There were total 41 cancer specific lncRNAs, the levels of which were also significantly different in clinical feature comparisons (P < 0.001, Table 2). Five lncRNAs (LINC01554, LOC255167, A1BG-AS1, LINC00526, and MIR22HG) were differentially expressed in three or four clinical feature comparisons.

**Table 2:** The correlations between cancer specific lncRNAs and clinical features. This table showed 41 cancer specific lncRNA which were also differentially expressed in clinical feature comparisons.

Comparisons	Down-regulated	Up-regulated
Gender (Female vs. Male)	LOC643837, LINC00526, FAM223B, MIR99AHG, LINC01554, TTTY15	H19, LINC00092, MIR600HG, LOC645676, COLCA1, LOC723809
Race (White vs. Asian)	GAS5, SNHG7, LOC100128191, SNHG1, HCG18	HCG11, LINC00242, GLIDR, LINC00261, LINC00638, LINC00574
Tumor grade (G3 + G4 vs. G1 + G2)	LOC255167, FAM99A, LINC00574, LOC399959, A1BG-AS1, LINC00526, LINC00261, MIR22HG, LOC643837	MALAT1, SNHG12, HCG18, SNHG20, ZFAS1, LOC440944, SNHG7, LOC100128191, MCM3AP-AS1, SNHG1, KCNQ1OT1, DSCR9, GAS5, LOC92659
AJCC TNM staging system (T3 + T4 vs. T1 + T2)	LOC255167, LINC01554, LINC01558, A1BG-AS1	MCM3AP-AS1
AJCC pathological stage (III + IV vs. I + II)	LOC255167, LINC01554, LINC01558, A1BG-AS1	UCA1
Vascular invasion (No vs. Yes)	MAPKAPK5-AS1, HNF1A-AS1, DSCR9	FAM99A
New tumor event (Yes vs. No)	MIR22HG	
Tumor status (With tumor vs. Tumor free)	MIR22HG	
Age at diagnosis (≥ 61 vs. < 61)		LINC01554, LINC00526, LOC255167

**Fig 1:**
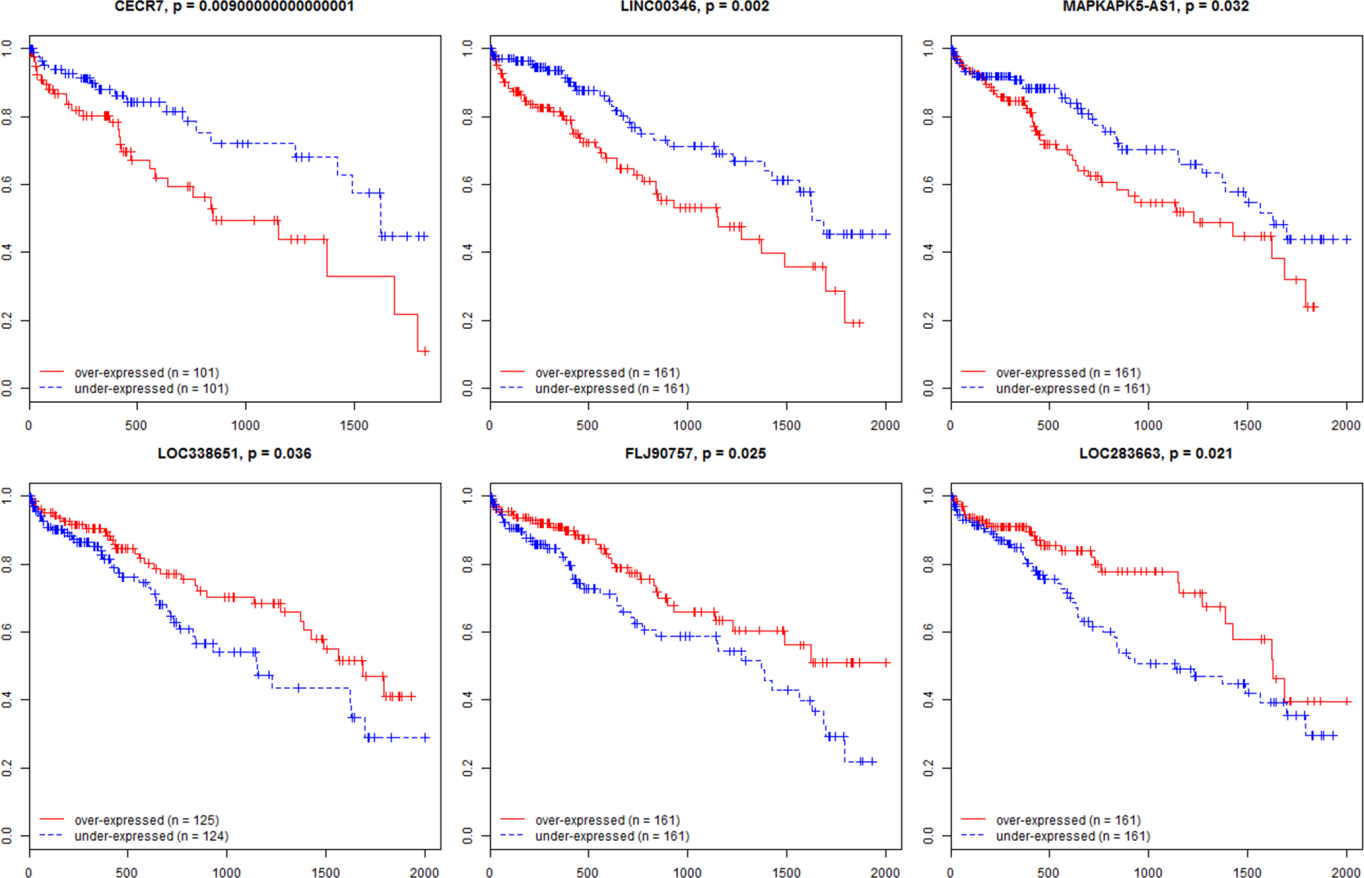
Kaplan-Meier survival curves for six lncRNAs associated with overall survival. (Horizontal axis: overall survival time: days, Vertical axis: survival function).

Subsequently, to identify the potential lncRNAs with prognostic characteristics, the levels of 177 lncRNAs in the TCGA data set were profiled using the univariate Cox proportional hazards regression model and six lncRNAs were found to be significantly associated with overall survival (log-rank P < 0.05). Among the six significant lncRNAs, three lncRNA (CECR7, LINC00346, and MAPKAPK5-AS1) were negatively associated with OS, while the remaining three (LOC338651, FLJ90757, and LOC283663) were positively correlated with OS (Fig 1).

### miRNA targeting lncRNAs predicted by miRcode and starBase

Our previous study has found 207 HCC-associated miRNAs which were differentially expressed between HCC tissues and adjacent non-tumor tissues [21]. We selected 33 miRNAs from 207 HCC-associated miRNAs in current TCGA data set (absolute fold change ≥ 3.0, P < 0.001, S2 Table). Here, we focused on whether these miRNAs would target above 28 cancer specific lncRNAs. In the ceRNA network, miRNAs interact with lncRNAs through MREs, we thus searched for the potential MREs in lncRNAs using miRcode [22] and starBase v2.0 [23]. The results showed that 17 of 33 cancer specific miRNAs might interact with 15 of 28 cancer specific lncRNAs (Table 3).

**Table 3:** Putative miRNAs that may target cancer specific lncRNAs by MREs. miR-199A-1 and miR-199A-2 were regards as a single miRNA in our study

lncRNA	miRNAs
LINC00176	miR-424, miR-184, miR-214, miR-93
PVT1	miR-195, miR-424, miR-183, miR-199A-1/2, miR-214, miR-383, miR-93
LINC00488	miR-139
RUSC1-AS1	miR-10B, miR-135A-1, miR-139, miR-182, miR-199A-1/2, miR-214, miR-96
FLVCR1-AS1	miR-375
GAS5	miR-10B, miR-139, miR-182, miR-490, miR-93, miR-96
UCA1	miR-214, miR-383
SNHG1	miR-182, miR-195, miR-383, miR-424
SNHG3	miR-135A-1, miR-139, miR-182
CDKN2B-AS1	miR-10B
LINC00152	miR-376C
CECR7	miR-199A-1/2, miR-214
DIO3OS	miR-10B, miR-139, miR-199A-1/2, miR-214, miR-34C
H19	miR-93
LINC00238	miR-33B, miR-375

### miRNA targets predicted by miRTarBase

To establish lncRNA-miRNA-mRNA network (ceRNA network), the next step was to search for mRNA targeted by miRNAs. Based on the miRNAs described in Table 3, we searched miRNA-targeted mRNA with experimental evidence using miRTarBase [24]. The results identified 17 miRNAs including miR-10B, miR-135A-1, miR-139, miR-182, miR-183, miR-184, miR-195, miR-199A-1/2, miR-214, miR-33B, miR-34C, miR-375, miR-376c, miR-383, miR-424, miR-93, and miR-96 (Table 4). Each miRNA-mRNA pair was experimentally validated by at least two of the following methods including reporter assay, western blot, qPCR, microarray, pSILAC (pulsed stable isotope labelling with amino acids in cell culture) or NGS (CLIP-seq or Degradome-seq). According to the allOnco database (http://www.bushmanlab.org/links/genelists), most of their targets are cancer-associated genes such as SIRT1, VEGFA, RASA1, RAF1, PTEN, MAPK9, MAPK8, MAPK1, MYC, MYB, KRAS, JAK2, IGF1R, IDH2, FOXO3, FOXO1, E2F3, E2F1, MAPK14, CDKN2A, CDKN1A, CDK6, CD44, CCNF, CCNE1, CCND3, CCND2, RUNX2, BCL2, CCND1, APC, AKT2, AKT1, ABCA1, etc.

**Table 4:** Validation of miRNA targets.

miRNA	mRNAs targeted by miRNAs
miR-10B	PPARA, NF1, CDKN2A, HOXD10, KLF4, NCOR2, CDKN1A, TFAP2C, BCL2L11
miR-135A-1	JAK2, NR3C2, APC, MYC
miR-139	FOS
miR-182	CDKN1A, FOXO1, MITF, FOXO3, RARG, ADCY6, CLOCK, TSC22D3, CYLD, BCL2, CCND2, PDCD4, RECK, EP300, FGF9, NTM
miR-183	SRSF2, PDCD4, AKAP12, FOXO1, ITGB1, KIF2A, BTRC, EZR, IDH2
miR-184	AKT2, INPPL1, NFATC2
miR-195	WEE1, E2F3, CDK6, BCL2, CCND1, RUNX2, RAF1, CCL4, BCL2L11, MECP2, CCND3, TBCCD1, VEGFA, SKI, KRT7, SLC2A3, CAB39, BCL2L2
miR-199A-1/2	CD44, IKBKB, MET, HIF1A, SMARCA2, SMARCA2, MAPK1, DDR1, MAP3K11, FUT4, CAV2, EZH2, CCNL1, MAPK9, AKT1, MAPK8, MAPK14, LIF, JUNB, MED6, MECP2, ETS2, EDN1, TMEM54, SIRT1
miR-214	PTEN, MAP2K3, MAPK8, PLXNB1, EZH2, POU4F2, GALNT7, XBP1, DAPK1, SRGAP1, TWIST1, BCL2L2, ING4, FLOT1, MAP2K5, HSPD1, AHSA1, CPNE7, RASA1, YWHAQ, ARL2, AP3B1
miR-33B	BCL2, ABCA1
miR-34C	MET, CDK4, NOTCH1, BCL2, E2F3, MYB, CAV1, CCNE2, MYCN, NOTCH4, ULBP2, MYC, SRSF2, SOX2, NANOG
miR-375	ELAVL4, YWHAZ, TIMM8A, PDK1, YAP1, MTDH, RASD1, YY1AP1, RHOA, KCNQ2, MTPN, USP1, JAK2, C1QBP, PLAG1, BCL2L11, RAB10, PARP1, CAB39, DLG4, ITGB1, SETD8, CASP3, CDC42, EIF2AK2, BCL2, NCAM1, LEPROTL1
miR-376C	ACVR1C, IGF1R
miR-383	DIO1, IRF1, VEGFA
miR-424	HIF1A, CUL2, SPI1, PLAG1, CCNE1, CCND3, CDK6, CCND1, MAP2K1, WEE1, NFIA, LGALS3, MYB, SIAH1, CCNF, CDC14A, CDC25A, CHEK1, KIF23, ATF6, ANLN, FGFR1, PIAS1, ITPR1
miR-93	CDKN1A, E2F1, ITGB8, TUSC2, TP53INP1, KAT2B, PTEN, LATS2, MAPK9, VEGFA
miR-96	CDKN1A, KRAS, FOXO1, FOXO3, ADCY6, REV1, RAD51, MITF, HTR1B, PRMT5

### ceRNA network construction and KEEG pathway analysis

**Fig 2:**
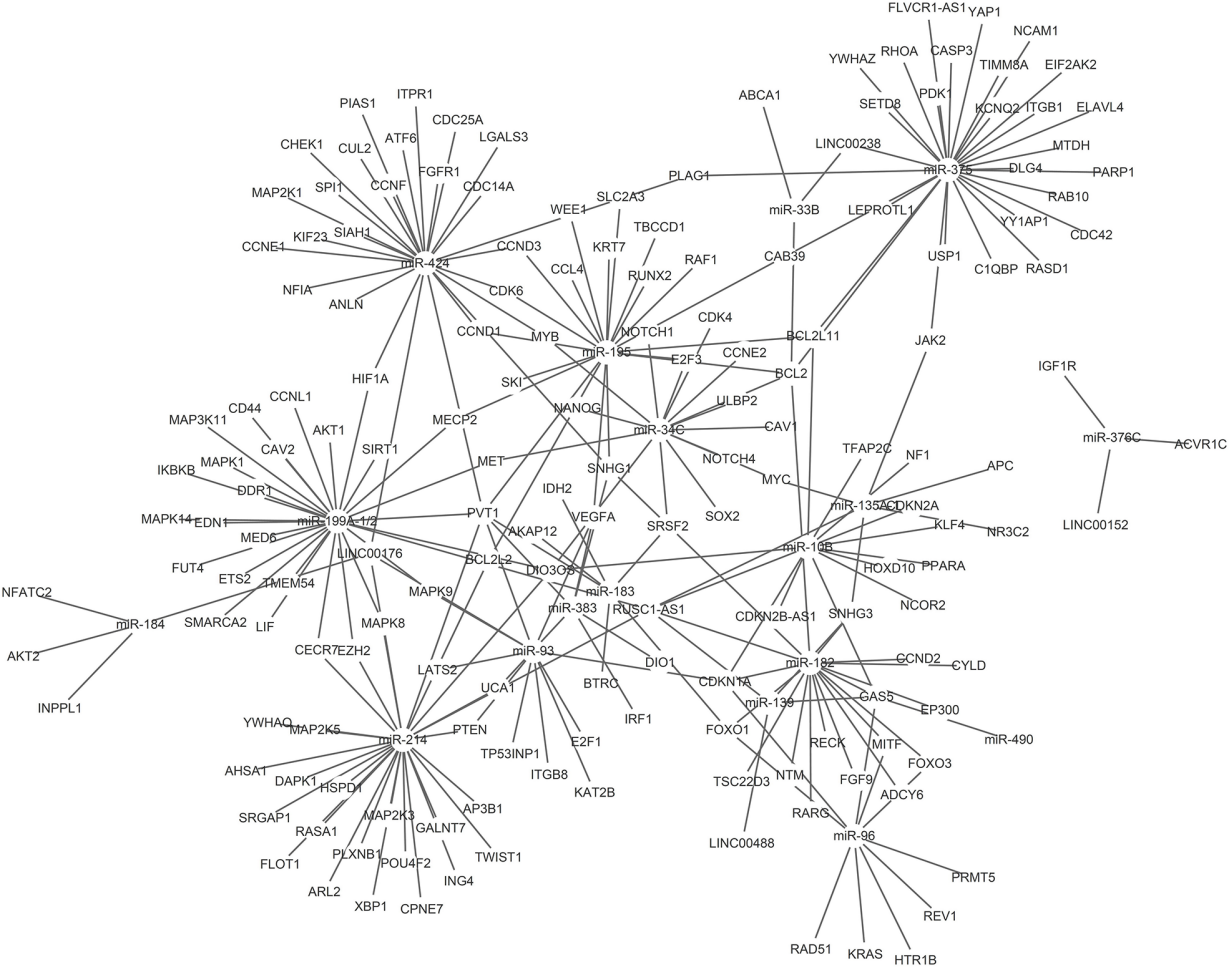
ceRNA network in HCC.

Based on the above data (Tables 3 and 4), we constructed a lncRNA-miRNA-mRNA ceRNA network. To get more robust results, we used the maximal information coefficient (MIC) algorithm to screen the pair-wised relationships based on the expression levels of the lncRNA, miRNA, and mRNA in TCGA data set (MIC > 0.15 and MIC-p^2^ > 0.15, please refer to Methods). 14 lncRNAs and 17 miRNAs were involved in the proposed ceRNA network (Fig 2).

In order to understand the signal pathways involved in ceRNA network, the mRNAs were analyzed by DAVID database. According to number of genes involved, we listed the top 15 KEGG pathways in our study (Table 5). Ten cancer-related pathways including pathways in cancer, pancreatic cancer, melanoma, prostate cancer, chronic myeloid leukemia, colorectal cancer, glioma, small cell lung cancer, bladder cancer, and non-small cell lung cancer, were enriched with the mRNAs, another 5 non-cancer related pathways such as MAPK signaling pathway, focal adhesion, cell cycle, neurotrophin signaling pathway, T cell receptor signaling pathway were also enriched.

**Table 5:** Top 15 KEEG pathways enriched by the coding genes involved in ceRNA network (P < 0.0001). The P value was corrected for multiple hypothesis testing using the Benjamini-Hochberg method (Please also refer to S3 Table)

	KEEG pathways	No. of Genes
Cancer related		
	Pathways in cancer	41
	Pancreatic cancer	19
	Melanoma	19
	Prostate cancer	19
	Chronic myeloid leukemia	16
	Colorectal cancer	16
	Glioma	15
	Small cell lung cancer	15
	Bladder cancer	13
	Non-small cell lung cancer	13
Non-cancer related		
	MAPK signaling pathway	22
	Focal adhesion	21
	Cell cycle	19
	Neurotrophin signaling pathway	18
	T cell receptor signaling pathway	14

## Discussion

Recently, lncRNAs have been emerged as abundant regulators of cell physiology in HCC and their functions may vary [25, 26]. Only a few studies have tried to reveal the lncRNA expression profiles in HCC by microarray with dozens of or even smaller sample size [13]. LncRNA and mRNA coexpression network was constructed by abnormally expressed lncRNA and mRNA [13]. A few studies described interactions between miRNA and lncRNAs [27, 28] or mRNA and lncRNA [29] in HCC, the results of which indicated that lncRNAs can function as a part of ceRNA network, but such ceRNA network is still poorly explored. In the present study, we identified tumor-specific lncRNAs in HCC and investigated their distributions in different clinical features and their associations with overall survival on the basis of genome-wide RNA profiles of 372 HCC tissues and 48 adjacent non-tumor liver tissues. Furthermore, we constructed a ceRNA network with cancer specific lncRNAs and miRNAs which provides a system biological views of lncRNA-miRNA-mRNA interactions.

Based on the next generation RNA sequence data from TCGA and GEO, we found that 177 cancer specific lncRNAs were differentially expressed in HCC tumor tissues and adjacent non-tumor liver tissues. Then, we revealed that 41 cancer specific lncRNAs were also abnormally expressed in different groups of clinical pathological features such as gender, race, tumor grade, AJCC TNM staging system, AJCC pathological stage, vascular invasion, new tumor event, tumor status, and age at diagnosis. Among the lncRNAs that differentially expressed in three or four groups, MIR22HG was reported to be an indicator of chemical stress responses in human-induced pluripotent stem cells [30]. We concluded that the expression of some lncRNAs is not equally distributed in certain situations. Future studies in this field should be properly designed to cope with this fact. Previous studies reported sexual disparity of HCC incidence [31], the differentially expressed lncRNA between female and male found in this study may contribute to this phenomenon. However, these unevenly distributed lncRNAs may not be significantly associated with overall survival.

With respect to the associations between cancer specific lncRNAs and patients’ survival, we found that six lncRNAs were related to HCC overall survival. Among the three risky lncRNAs, CECR7 is a candidate lncRNA for Cat Eye Syndrome [32]. The functions of the other two risky and three protective novel lncRNAs are still unclear. It is also noted that five of these six lncRNAs (CECR7, LINC00346, LOC338651, FLJ90757, and LOC283663) were not differentially expressed in any clinical feature comparison. Therefore, lncRNAs that do not differentially express in clinical feature comparisons can be correlated with overall survival, whereas lncRNAs that differentially express in clinical feature comparisons may not be necessary to be associated with overall survival.

We believe that there may be some cross-talks between lncRNA, miRNA and mRNA in the progress of HCC. We applied several steps to increase the accuracy of ceRNA network prediction. First, we only included the cancer specific lncRNAs and miRNAs that had an absolute fold change ≥ 3.0 and was annotated by GENCODE. Second, the relationships between lncRNA and miRNA, and miRNA and mRNA were predicted by experiment- supported algorithms or databases such as miRcode, starBase and miRTarBase. These two measurements ensured that the relationships identified would occur not only *in silico* situations but also by experimental-supported evidences.

To further improve the performance of our prediction, the maximal information coefficient (MIC) algorithm and maximal information-based nonparametric exploration (MINE) statistics were used to filter the pair-wised relationships based on lncRNA-miRNA-mRNA expression correlations. In general gene co-expression network analysis, Pearson’s correlation is a measure for linear regression, but it is very sensitive to outliers. MIC and MINE are able to examine and characterize all potentially interesting relationships in a complex data set [33].

The ceRNA network we built brings to light an unknown ceRNA regulatory network in HCC. In this newly-identified ceRNA network, many oncogenes and tumor suppressors participate in HCC development and treatments. A recent study also identified that lncRNA-miRNA-mRNA interactions were active and might act as potential prognostic biomarkers in cancers [34].

In conclusion, our study has found the cancer specific lncRNAs in HCC using hundreds of candidate lncRNAs and large scale samples, and disclosed abnormal expression pattern of cancer specific lncRNAs under different clinical features. Importantly, we have constructed a ceRNA network to propose a new approach to lncRNA research in HCC. Our findings suggest that cancer specific lncRNAs in HCC may participate in a complex ceRNA network.

## Supporting Information

S1 Table 177 and 37 differentially expressed lncRNAs between HCC tissues and adjacent non-tumor tissues in TCGA data set and GEO data set. (XLS)

S2 Table 33 HCC-associated miRNAs between HCC tissues and adjacent non-tumor tissues in TCGA data set. (XLS)

S3 Table All KEEG pathways enriched by the coding genes involved in ceRNA network (P < 0.05). (XLS)
